# ‘*I perceive it to be less harmful, I have no idea if it is or not:*’ a qualitative exploration of the harm perceptions of IQOS among adult users

**DOI:** 10.1186/s12954-021-00490-8

**Published:** 2021-04-13

**Authors:** Katherine A. East, Charlotte N. E. Tompkins, Ann McNeill, Sara C. Hitchman

**Affiliations:** 1 National Addiction Centre, Institute of Psychiatry, Psychology and Neuroscience, King’s College London, London, UK; 2 School of Public Health and Health Systems, Faculty of Applied Health Sciences, University of Waterloo, Waterloo, Canada; 3 Shaping Public hEalth poliCies To Reduce ineqUalities and harM (SPECTRUM), Edinburgh, UK

**Keywords:** Tobacco, Nicotine, Harm perceptions, Qualitative research

## Abstract

**Background:**

Harm perceptions of tobacco and nicotine products can influence their use and could be targeted by policies to change behaviour. IQOS was introduced to the UK in 2016, and there is little independent qualitative research on IQOS harm perceptions. This study explored the perceived health harms of IQOS to users and those exposed to the emissions, what shapes these perceptions, and what participants wanted to know about the harms of IQOS.

**Methods:**

Qualitative interviews in London, UK, with 30 adult current and former IQOS users who currently smoked or quit smoking in the last 2 years.

**Results:**

IQOS was perceived as less harmful than smoking but not risk-free, although there was great uncertainty. Influences on harm perceptions were consolidated into six themes: *(1) dominance of manufacturer claims* influenced perceptions that IQOS is less harmful than smoking to users and those around them, although mistrust of the tobacco industry heightened scepticism about harms; *(2) limited independent and long-term research* led to uncertainty about harms, although some participants trusted IQOS would not be marketed if it were very harmful. Participants wanted more independent and long-term studies into harm; *(3) appearance of HEETS (tobacco sticks) packaging* conveyed reduced harm because packets were ‘*pretty*’, without graphic/specific warnings, although written warnings conveyed some harm. Participants wanted more information on HEETS packets about harms; *(4) process of heating and HEETS contents*—heating, compared with burning, tobacco was perceived to produce fewer harmful chemicals, while tobacco, nicotine, and chemicals in HEETS were perceived to cause some harm. Participants wanted clarification about the harms of heating tobacco and HEETS ingredients; *(5) improvements in physical health and personal appearance* reduced perceptions of harm*; (6) differences in sensory experiences* (taste, sight, smell) when using IQOS over smoking reduced perceptions of harm, while ‘black’ deposits inside IQOS led to perceptions of some harm. Reduced volume and smell of IQOS emissions also reduced perceptions of harm to non-users exposed to the emissions.

**Conclusions:**

IQOS was perceived as less harmful than smoking but not risk-free, although there was great uncertainty. Participants wanted clarification about IQOS harms from independent sources in accessible forms, specifically related to HEETS ingredients, heating tobacco, and emissions to others.

## Introduction

**Fig. 1:**
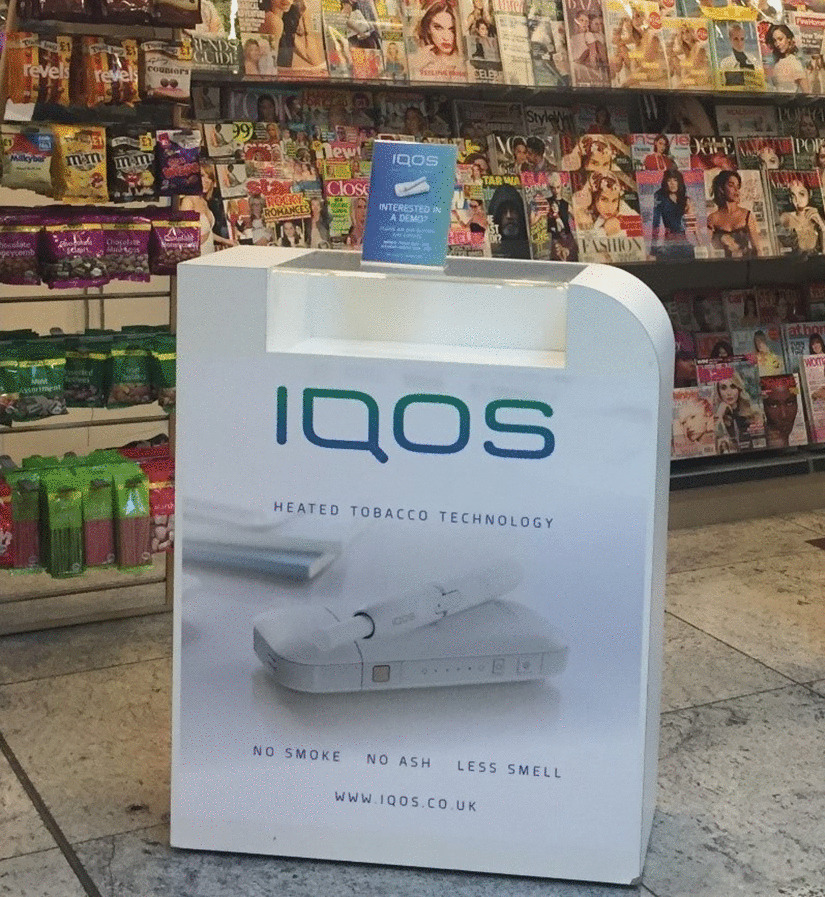
IQOS advertising in a UK newsagent

Heated Tobacco Products (HTPs) are electronic devices that typically heat tobacco to generate an aerosol that is inhaled. IQOS is a HTP manufactured by Philip Morris International (PMI), who also manufacture Marlboro cigarettes. IQOS is the most widely available HTP, available in over 45 countries globally, of which at least 20 are in Europe [1, 2]. In the UK, PMI specifically advertise IQOS as ‘a new alternative to smoking that heats tobacco rather than burning it’, [3] which produces ‘no smoke’, ‘no ash’ and ‘less smell’ (Fig. 1) and ‘produces 95% less harmful chemicals compared to cigarettes’ [3].

Independent reviews of HTPs including IQOS have found that evidence for reduced production of tobacco-specific harmful chemicals is predominantly funded by manufacturers [4–6]. Some independent studies have also reported that the production of some harmful chemicals is lower for HTPs [4, 6], and IQOS specifically [7–10], than tobacco cigarettes, while other studies have reported the presence of substances with unknown harms to health [11]. In July 2020, the US Food and Drug Administration (FDA) concluded that switching completely from conventional cigarette smoking to using IQOS specifically reduces exposure to harmful or potentially harmful chemicals [12, 13]. However, the FDA did not find sufficient evidence to accept PMI’s claims that IQOS use reduced the risks of tobacco-related diseases or risk of harm relative to continuing to smoke cigarettes [12, 13]. That is: PMI’s marketing claims of reduced exposure to harmful chemicals were accepted, while claims of reduced risks of tobacco-related diseases or harm were not.

Self-report surveys from Germany [14], Japan [15], and Switzerland [16] indicate that HTPs (and IQOS specifically [16]) are generally perceived to be less harmful [14, 15] or less risky to health [16] relative to smoking combustible cigarettes, as opposed to equally or more harmful/risky. However, there is a high degree of uncertainty: the only one of these surveys to report ‘don’t know’ responses found that over a quarter (26%) of respondents did not know the harms of HTPs relative to smoking [15].

Understanding the health harm perceptions of HTPs is important because harm perceptions of nicotine and tobacco products can influence their use [17–22] and hence could be targeted by policies and interventions to change HTP use and/or smoking. For example, adult smokers and ex-smokers who perceive electronic cigarettes (e-cigarettes) to be less harmful than smoking are more likely to try or currently use an e-cigarette than those who do not share this view [17–20]. Similarly, perceptions of reduced harm relative to smoking are among the most commonly reported reasons for choosing a specific HTP brand, including IQOS, in Japan (a country with a number of HTPs and one of the first to launch IQOS) [23] as well as for using IQOS specifically in a study involving users from several countries [16]. Perceptions of reduced harm relative to smoking have also been associated with frequency of IQOS use in Japan [15].

Harm perceptions of nicotine and tobacco products can be influenced by several factors, including reduced-risk claims [24], manufacturer advertisements [25], and the appearance of packaging including health warnings [26–29]. There is little independent research on what influences harm perceptions of HTPs specifically, although quantitative surveys in Japan [15] and experimental data from the US [30] indicate that advertisements and reduced-risk claims promote views that HTPs are less harmful than smoking. These studies did not explore what else may influence harm perceptions of IQOS specifically, what influences perceptions that they are more harmful, equally harmful or uncertainty about harms, nor harm perceptions of IQOS to the user relative to second-hand emissions to non-users.

Qualitative research can help address these gaps by exploring peoples’ health harm perceptions in depth and unpacking the different aspects of harm perceptions, how and why they are formed, and how they might be susceptible to change. For example, a recent qualitative focus group study in South Korea found that users and ex-users of HTPs perceived HTPs, although not IQOS specifically, to be less harmful than smoking because of the difference in smell and a reduction of sputum and tartar, while the tobacco content of HTPs led some to perceive HTPs to be equally as harmful as smoking [31]. Findings from such qualitative studies can also help to understand how to communicate the health harms of HTPs, and inform the design and improve the validity of quantitative survey measures.

**Fig. 2:**
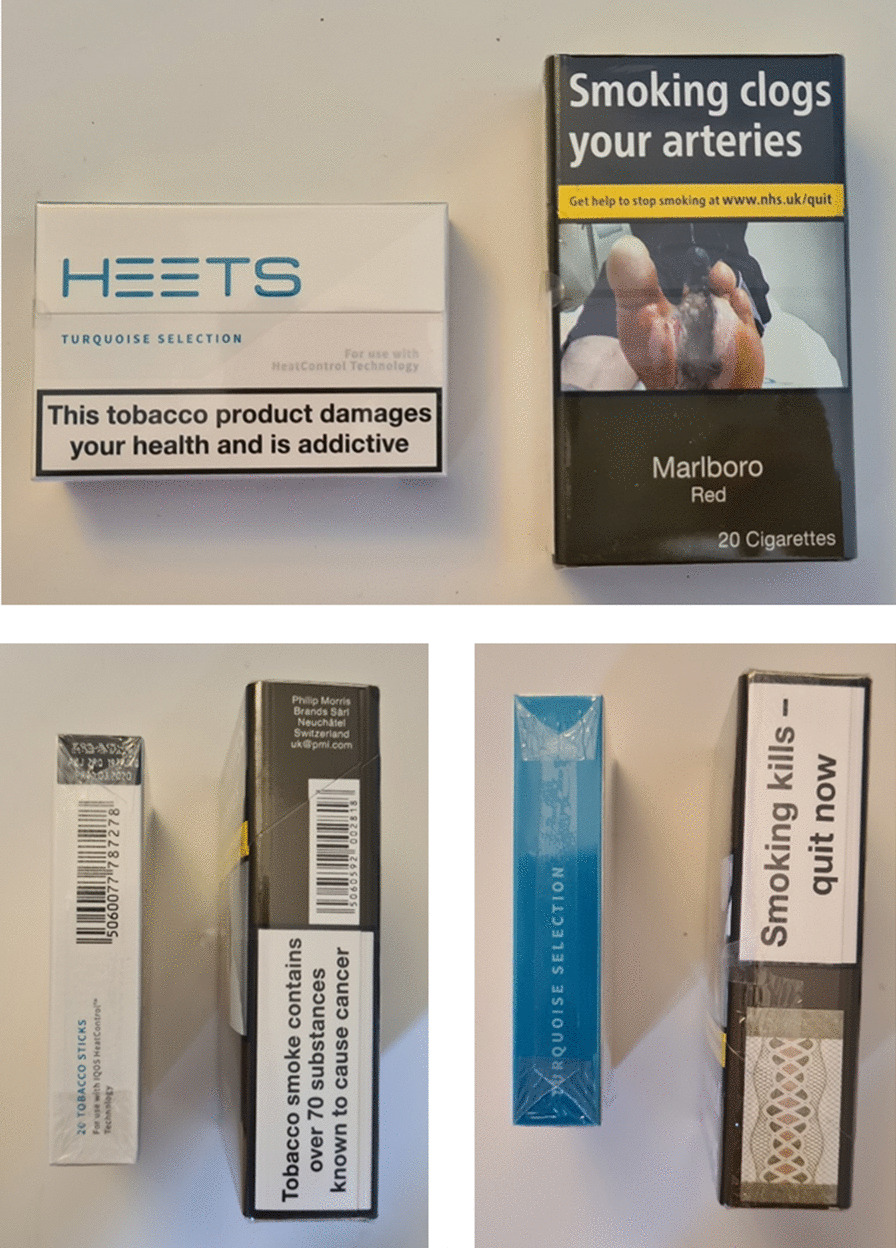
HEETS tobacco sticks packet (left) and combustible cigarette packet (right) in the UK. Top figure = front view of packets. Bottom figures = side views of packets

IQOS was introduced to the UK market in 2016, although prevalence of IQOS use in the UK is uncertain [32]. Between 2018 and 2019 we conducted qualitative research to explore use of IQOS among current and former users in London, UK, using one-to-one interviews [33]. At the time of this study, advertising was present for the IQOS device (Fig. 1) but not HEETS (tobacco sticks used with the IQOS device), and unlike combustible cigarette packaging which had to be standardised (plain) by May 2017, HTPs were not subject to standardised packaging or pictorial health warnings, although text warnings covering 30% of HEETS packets were mandated (Fig. 2). We previously reported that, among other factors, initiation and continued use of IQOS were influenced by perceiving IQOS as less harmful to users’ health than smoking combustible cigarettes. Reduced-risk marketing claims, ‘cleaner’ packaging of HEETS with less alarming health warnings, and perceived improvements in users’ personal physical health after switching to IQOS contributed to these views. Conversely, IQOS use was discouraged by concerns about unknown harms, due to the perceived lack of independent and longitudinal research, PMI disclaimers, written warnings on HEETS packaging, and the contents of HEETS [33]. However, our previous analyses and publication focussed on what influenced IQOS *use*, and did not consider or explore the health harm perceptions of IQOS in any detail other than their role in influencing use.

In this paper, we build on our previous findings to specifically explore the perceived health harms of using IQOS. We focus on the perceived health harms to individual users as well as the emissions to those around them and unpack what shapes these perceptions and in what way. We also identify what participants still wanted to know about the potential health harms of using IQOS and their preferences for receiving this information.

## Methods

Our methods are described in detail elsewhere [33]. Briefly, to inform the research focus and methods, we first consulted seven international tobacco control experts and a national panel of twelve current and former smokers. We aimed to interview a range of participants in terms of their demographics and smoking/IQOS use history; those interested in taking part were purposefully sampled based on this information. We approached individuals using IQOS in public, advertised the study online and in London vape shops, and monitored the sample characteristics throughout recruitment. All study materials and approaches to potential participants clearly stated that we were conducting independent, academic research. Adults age 18 + years were eligible to take part if they were resident in the UK, currently *or* formerly used IQOS at least weekly for at least one month, and currently or formerly smoked (quit in the last 2 years).

This study received ethical approval by King’s College London Research Ethics Committee (LRS-17/18-5765), and participants were assured of their confidentiality and provided written informed consent. Thirty current and former IQOS users were interviewed one-to-one over a five-month period between 2018 and 2019. Interviews lasted a mean of 67 min, were audio recorded, and transcribed verbatim. Participants were offered a £20 shopping voucher for taking part.

A topic guide directed the interviews, which covered participants’ smoking history, experiences of using IQOS, perceptions of health harms for users and second-hand emissions to non-users, sources of health harm-related information, and what participants still wanted to know about the health harms of using IQOS. An open and flexible questioning style was used, to be responsive to participants. For example, if participants naturally introduced views on the harms of IQOS, their responses were probed in full by asking questions such as ‘why do you say that?’ and ‘where did you get this information from?’. If harm perceptions were not introduced naturally by participants, the interviewer introduced the topic openly, such as ‘what, if any, do you perceive are the potential harms of IQOS’, and responses were probed.

Data coding and analyses were guided by Iterative Categorization [34], a systematic and staged approach to qualitative data management and analyses. First, we read a selection of interviews and developed a coding frame. Then, we imported the coding frame and the transcripts into MaxQDA—a software package to support qualitative data coding and analysis—and coded all text in each transcript to the relevant code(s) [35]. For this paper, we systematically reviewed the data within the ‘harm perceptions’ code. During this process, we grouped the data into smaller sub-codes (e.g. ‘concerns and uncertainties’, ‘lack of evidence’, ‘harm to others’) and examined the data for similarities and differences, such as between current and former IQOS users. From there, we inductively consolidated the harm perceptions sub-codes and re-organised them at a higher level of abstraction according to what shaped participants’ views on the health harms of IQOS. This process identified six overarching themes: (1) dominance of manufacturer claims; (2) limited independent and long-term research; (3) appearance of HEETS packaging; (4) process of heating and HEETS contents; (5) improvements in physical health and personal appearance; and (6) differences in sensory experiences.

## Findings

### Sample

Participants were all young adults or middle aged (modal age 35–49; Table 1). Most were White British or White Other (including European), in managerial or professional occupations, and were, or had been, self-defined users of tobacco or nicotine products for at least 6 years (Table 1). Most were current IQOS users, and half of all participants were current IQOS users who also smoked currently (Table 1).

**Table 1:** Sample characteristics (*n* = 30)

	*n* in sample
*Age (years)*	
18–24	7
25–34	8
35–49	12
50–59	3
*Gender*	
Male	19
Female	11
*Ethnicity*	
White British	8
White Other	17
Asian British	3
Black British	1
Arabic	1
*Occupation*	
Professional/qualified	9
Managerial/senior administrator	9
Clerical/junior administrator	8
Sales/services	1
Semi-skilled/unskilled labour	1
Never worked	2
*Patterns of current IQOS use and smoking*	
Current IQOS user	22
(Current smoking—daily/weekly/monthly/less than monthly)	(15)
(Current smoking—not at all)	(7)
Former IQOS user	8
(Current smoking—daily/weekly/monthly/less than monthly)	(6)
(Current smoking—not at all)	(2)
*Length of IQOS use (months)*	
1–3	4
4–6	7
7–12	11
More than 12	8
*Current frequency of cigarette smoking*	
Daily	7
Weekly	4
Monthly	4
Less than monthly	6
Not at all	9
*Time using tobacco and nicotine products*^*a*^	
1–5 years	9
6–10 years	5
11–20 years	3
More than 20 years	12

^a^Includes use of other tobacco and nicotine products (e.g. shisha and cigars). One participant did not disclose time since using tobacco and nicotine products, so missing data exist

### Overall perceptions of harm

Most participants perceived that IQOS was ‘*less harmful*’ or ‘*better’* for their health than smoking combustible cigarettes but also acknowledged that there must be ‘*some harm*’ from using IQOS, and that it would be *‘better’* for their health to not use IQOS at all. However, our detailed analyses of the harm perceptions data identified that participants had nuanced views which were frequently veiled in uncertainty, with participants remaining cautious about the potential health consequences of IQOS, stating that it was *‘possibly safer, but just as possibly not*’.

We now report the six themes that shaped participants’ perceptions of the health harms of using IQOS, illustrated using verbatim quotations labelled with pseudonyms to protect anonymity. Our analyses did not identify any notable differences between participants, including current and former IQOS users.

### Dominance of manufacturer claims

Several participants recalled manufacturer claims, some from ‘*PMI’* specifically, that IQOS is *‘90% healthier’, ‘90% less hazardous’, ‘90% better’, ‘90% safer’* or*’90–95% less harmful’* for users than smoking, which strongly underpinned their beliefs about the reduced health harms.

*The lady* [PMI IQOS sales rep] *who was promoting the IQOS, she was very convincing… she assured me that it was safer than cigarettes.* (Alison, age 35)

Some participants interpreted PMI’s specific reduced-risk claims as a reduced risk or ‘*fraction’* of the risk of developing smoking-related ‘*diseases’* and *‘cancers’* when using IQOS compared with smoking*.* However, others interpreted PMI’s claims as there still being ‘some harm’.

*Even if Philip Morris is saying they’re 90% healthier, there’s still a 10% harm* (Sean, age 52)

Some recalled PMI’s claims that there is *‘no smoke,’ ‘no second-hand smoke’* from IQOS or that any ‘*second-hand smoke*’ is *‘not harmful’* to others, contrasting with the known harms of passive smoking.

*Philip Morris claim that this you can smoke indoors and people living in the house will not be affected and it’s safe*. (Francesca, age 46)

However, participants raised ‘*an element of doubt’* or ‘*healthy scepticism’* about relying on information from PMI about the health harms of using IQOS for users and second-hand emissions to non-users because they had a general mistrust of *‘big tobacco’* which stemmed from the industry’s ‘*history of being very, very sly and deceitful’* and poor *‘track record’* of disclosing the health harms of smoking*.*

*Philip Morris have* *lied to us before about cigarettes... what’s to say they’re not lying about the IQOS?... No one trusts Philip Morris... they’re a corporation trying to make money… they’ve obviously promoted smoking, which is deadly.* (William, age 36)

Consequently, they questioned whether PMI created IQOS because *‘it’s better*’ than combustible cigarettes or to protect their business interest and remain profitable and ‘*keep the customer smoking*’ to combat the ‘*shift’* away from combustible cigarettes.

Doubts regarding PMI’s impartiality underpinned participants’ hesitation to trust any affiliated research into IQOS. They considered that researchers may have received a *‘pay off’* from PMI to *‘push’* IQOS *‘to market’* and expressed concerns that PMI only presented ‘*positive*’ findings from their research.

*I* *just don’t trust them *[PMI] *to be 100% objective or not to spin or interpret data in a way that suits their commercial objectives*. (Sanjay, age 43)

Adding to their mistrust, some participants believed that PMI had heavily criticised or ‘*managed to squash*’ others’ research with *‘significantly different’* findings to their own. Yet, participants were also wary of IQOS-related research or claims from ‘*rival’* tobacco companies and speculated that they may use ‘*bad tactics’* to *‘look for flaws’* or *‘skew’* findings to discredit PMI and protect their own business.

At the same time, individuals conceded that PMI were unlikely to ‘*gamble’* or jeopardise their business by *‘publishing falsified information’.* They also acknowledged having seen and accepted disclaimers on the PMI website or in IQOS stores stating that IQOS *‘is not a health product’* and that using it is *‘bad for you’* and *‘not without risk’.*

As a consequence of these claims and beliefs, participants chose to ‘*hope’,* ‘*pretend,*’ *‘fool’,* or ‘*convince’* themselves that using IQOS is indeed less harmful for their health or *‘damaging me less’* than smoking combustible cigarettes. Yet, their narratives simultaneously highlighted their overall uncertainty as they acknowledged that their views were *‘optimistic’, ‘an illusion’* or akin to *‘living in a fairy-tale’.*

*I just hope and pray that what is claimed is what it is… it’s 90% safer for you than smoking… I would be incredibly annoyed… if… the claims they made were utter rubbish.* (Neal, age 47)

Reflecting this uncertainty, participants wanted further investigations to identify how ‘*reliable’* and ‘*conclusive’* PMI’s research and claims about the health consequences of using IQOS are for users and also for non-users from second-hand emissions.

*What’s it doing to my health?… Philip Morris’ headline is 90 to 95% better than smoking … is for your health. So is that correct? That’s what I want to know. *(William, age 36).

### Limited independent and long-term research

Some participants were uncertain about the potential health harms of using IQOS after their attempts to find relevant information online via regular search engines revealed a lack of independent research.

*I was hoping to find independent research or whatever, but there’s nothing.* (Darius, age 30)

Others were confused and ‘*afraid’* that IQOS could be ‘*as harmful,*’ or ‘*more harmful*’ or ‘*worse*’ than smoking after they had found ‘*no consensus on how much, if any, better they were for you than cigarettes’,* or information that seemed inconclusive or in conflict with their interpretation of PMI’s claims.

Participants suggested that the lack of independent and long-term research and ‘*testing’* was because IQOS was *‘new’,* ‘*fresh to the market,*’ and had not ‘*been out long enough*’ and hence there were ‘*too many unknowns*’. While they lamented the lack of independent information, particularly about the long-term health harms of using IQOS, they conceded that it would take many years for research to identify any causal links because cancer and other smoking-related diseases can take years to develop.

In the absence of readily available independent information, participants speculated that IQOS could lead to a range of potential health outcomes such as arterial, cardiovascular and lung diseases, cancers or even ‘*new’* diseases.

*I just don’t think it’s been out long enough… this isn’t something that you can just study in a year or two. So five years later someone could get, god forbid, some kind of weird new disease around something IQOS has produced… it’s way too early to claim this is 100% what it says.* (Yusuf, age 25)

Despite the perceived lack of independent research, participants trusted that IQOS would not have been allowed to be marketed ‘*without it being thoroughly tested*’ or if it were ‘*very dangerous*’ for users’ health.

*One has to have some element of trust in the UK authorities that it’s been licensed… that I’m not going to drop dead… from using it.* (Clive, age 59).

Nevertheless, participants overwhelmingly wanted ‘*more research*’ and ‘*further trials*’, especially from ‘*independent’* sources to clarify the *‘potential harms’* and long-term health consequences of using IQOS to both themselves and exposure to emissions among non-users including co-residents, family members, partners, as well as members of the public.

They specifically mentioned that seeing information about the health harms of IQOS from public bodies, such as the NHS and from respected media channels or independent cancer charities would increase their confidence in any research conducted and associated claims.

*I’d be really interested to see any research that I’ve missed on the health risks of IQOS… that information needs to be more commonly available, because at the moment there’s just not enough out there.* (Peter, age 39)

#### Appearance of HEETS packaging

Overwhelmingly, participants believed that IQOS was less harmful than smoking because of the appearance of HEETS packets. As well as ‘*clean’,* participants commented that the packets were ‘*nicely designed,*’ ‘*appealing*’ and ‘*pretty’* compared to the unbranded, standardised ‘*greenish’* colour of combustible cigarette and rolling tobacco packets on the UK market (Fig. 2).

While the health warnings on HEETS packets served the purpose of conveying some harm, participants considered the harm was less than from smoking combustible cigarettes because the ‘*warnings’* were less severe and they did not detail specific diseases like combustible cigarette and rolling tobacco packets (Fig. 2).

*It *[HEETS packet] *says, ‘this tobacco product damages your health and is addictive.’ OK, well I’ve never seen ‘causes heart disease, causes lung cancer,’ never seen ‘may cause death’—you’ve got that on cigarette packets.* (Karina, age 22).

Furthermore, participants assumed that IQOS was less harmful than smoking because HEETS packets did not feature any *‘horrible’* or *‘disgusting’* photographic images of the potential diseases caused by using them. Indeed, they commented that unlike combustible cigarettes, photographs of mouth cancer, ‘*diseased’, ‘exploded’,* or *‘black’* lungs, *‘rotting feet’* or *‘ill babies’* were absent from the packets.

At the same time, some questioned whether the lack of written and visual warnings was genuinely attributable to the reduced harms of using IQOS. Instead, they speculated whether insufficient evidence about the health risks, the limited ‘*space’* on the ‘*small’* packets or a lack of mandatory regulations explained the lesser warnings. These speculations contributed to concerns that the health consequences of using IQOS remained unknown or undisclosed.

*I don’t know if it doesn’t have harmful pictures because it doesn’t have space… or because it’s not that harmful… if they’re selling the idea that it’s not as harmful as regular cigarettes, they wouldn’t put* [warning images on HEETS packets]… *they don’t want to sell that idea.* (Elena, age 25)

Consequently, some participants suggested that HEETS packets should contain more information about the associated health harms.

*Erring on the side of caution, they* [HEETS packets] *should probably carry the same or similar warnings* [as combustible cigarette packets]*… because inevitably, either subliminally or explicitly, people will think they’re less harmful if they don’t.* (Sanjay, age 43)

### Process of heating and HEETS contents

In contrast with burning combustible cigarettes, some participants said that heating tobacco prevented combustion and thus produced ‘*fewer harmful chemicals*’ and less tar, carbon monoxide, carcinogens, and other disease-causing substances.

*If you burn something… it produces carbon monoxide, which is obviously a very harmful substance, because it binds to your red blood cells in preference to oxygen, and that’s what like damages your lungs… that’s not what happens in IQOS, because you just heat it up, you don’t burn it… it’s less harmful.* (Nicholas, age 18)

The perceived risk of developing specific diseases was influenced by the perceived reduction in tar produced when avoiding combustion.

*There’s no tar… lesser risk of lung and throat and mouth diseases…the lips cancer, tongue cancer, throat cancer, lung cancer.* (Darius, age 30)

Yet, a few participants believed that the specific health harms of heating tobacco were unknown and they wanted to know more about this.

*Everyone says about the heating against burning, but it’s something no one knows what actually does to your body… we know cigarettes are bad… but heating, we have no idea what it does.* (Marco, age 23).

Perceptions of harm were also shaped by the contents of HEETS. Most commonly, participants felt that they lacked information about the exact contents and composition of HEETS. This led to concern that using IQOS could have ‘*any sort of side effects*’, be as harmful or more harmful than smoking, or could cause ‘*new’,* ‘*additional,*’ or ‘*unknown’* health issues.

*It’s a new kind of formulation of tobacco… it’s hard to be sure what additional damage that could cause, beyond the bits they are already known of from cigarettes*. (Raj, age 43)

When describing HEETS, participants referred to ‘*tobacco’,* ‘*chemicals’,* and ‘*nicotine’,* and as a result expected IQOS use would result in some harm, specifically ‘*some kind of disease*’ or ‘*a certain amount of damage to your lungs.*’ A few also thought that HEETS contained fewer ‘*chemicals*’ or ‘*additives*’ than combustible cigarettes, leading them to believe that using IQOS was less harmful to their health.

Nicotine was perceived as a source of *‘some harm’,* specifically ‘*addiction’*, and ‘*nefarious effects’* such as ‘*cardiovascular disease’* and *‘denser blood’.* Yet, participants reported that they were unaware of the nicotine content of HEETS because this was not stated on the packets, and so they questioned the content and their nicotine intake when using IQOS, and potential associated harms. Consequently, participants wanted to know more from PMI about the specific ingredients of HEETS and the amount of nicotine that HEETS contain to help them understand the potential harms from using IQOS.

*I want to know the amount of nicotine, what is in one HEET, and I want to know if… they can start producing the HEETS with little amount of nicotine, so… you can choose, for example when I go to buy a coffee I can choose the light one, not strong.* (Martyna, age 37).

### Improvements in physical health and personal appearance

Participants reported noticing improvements in their own physical health soon after using IQOS compared to smoking. This contributed to their perceptions that using IQOS is less harmful or *‘less unhealthy’* than combustible cigarettes, at least in the short term. Specifically, they felt ‘*fitter’* or ‘*healthier’* overall, they had more ‘*stamina*’ during physical exertion, and their respiratory health improved and their experience of respiratory conditions lessened as their lungs and chest were less ‘*tight,*’ ‘*heavy,*’ or ‘*clogged up.*’.

*After a short period of time… I was coughing less and I had better cardiovascular breathing ability during exercise, and so this for me suggested that OK, that it may not be good, but it is better* [than smoking]. (Daniel, age 49)

Improvements in their personal physical appearance, such as *‘clearer skin*’ and less staining on teeth and fingers further added to participants’ impressions that there was less harm associated with IQOS use compared to smoking.

*I think it’s got to be less harmful… over a period of five or six months when I was using it* [IQOS]*…* *I felt better… people around me said you look much better, are you training? And I said no, I’ve actually stopped smoking…my cheeks were fuller and redder… I looked better than I did when I smoked.* (Hayden, age 48).

Despite noticing these immediate improvements, some participants were unclear about whether these improvements would remain in the long term and they wanted to know what, if any, long-term physical health consequences were associated with using IQOS (e.g. ‘*cancers’, ‘lung disease’, ‘cardiovascular disease’).*

### Differences in sensory experiences

Contributing to their perceptions that IQOS was less harmful, participants reported key sensory differences (e.g. taste, sight, and smell of emissions) when using IQOS compared with smoking combustible cigarettes. That is, in addition to tasting ‘*lighter’* and less ‘*harsh’,* IQOS also felt less ‘*hot*’ in the mouth and on the throat.

*It feels like smoking, but it doesn’t feel like you’re getting the nasty effects… Using a normal cigarette, I’m like… causing cancer in the back of my mouth... Whereas with the IQOS, it doesn’t feel like that… my throat doesn’t feel sore and my breathing doesn’t seem to be affected.* (Maria, age 29)

Visually, participants reported that the ‘*smoke’* exhaled when using IQOS looked *‘cleaner’, ‘more transparent’,* and ‘*less black*’ than combustible cigarette smoke, leading to perceptions of reduced harm. They also noted that IQOS emissions appeared *‘less dense’* and dispersed more quickly.

*It does appear to be more healthy… With a normal cigarette… you can imagine your lungs getting darker with that smoke. Whereas with IQOS… it looks cleaner… the smoke is much more transparent, much more vapour-like than actual smoke.* (Miguel, age 26)

Additionally, the reduced smell produced when using IQOS compared to combustible cigarettes reinforced perceptions of reduced harm.

*The fact that you don’t stink after it is an even greater presumption that it’s doing you less damage.* (Max, age 41)

The reduced volume, appearance, and smell of the ‘*smoke’* produced led some participants to believe that using IQOS was also less harmful to non-smokers exposed to the emissions, including partners, children, co-residents, and pets. However, while such beliefs made participants feel more comfortable about using IQOS around non-smokers, others expressed reservations about the possible health risks of ‘*second-hand smoke*’ from IQOS, such as ‘*some kind of nasty disease’,* and welcomed clarification about these.

[IQOS] *seems to be a healthier alternative, not just for me but the people around me… but I guess I would need to know for sure that there wasn’t any dangers around the second-hand smoking or using of HEETS* (Sean, age 52)

Participants expressed mixed views about the appearance of used HEETS filters and residue inside the IQOS device. Some distinguished that the filters of used HEETS appeared *‘cleaner’* and less ‘*tarred’, ‘stained*’, or ‘*yellow’* than used cigarette filters and thus perceived using IQOS to be less harmful. Yet others raised concerns that there may be health harms from inhaling the *‘black’,* ‘*disgusting*’ tobacco ‘*residue’* which amassed inside the IQOS device if it was not cleaned regularly between uses.

*The little piece of metal that’s meant to heat the tobacco… some tobacco usually gets stuck. It’s quite nasty, because it turns black over time, which kind of makes you think* *about what you have on your lungs.* (Yasmina, age 25)

## Discussion

This paper expands our previous publication [33] using data from in-depth interviews by exploring health harm perceptions of IQOS among current and former users and identifying what shapes these perceptions and in what way. Our sample appeared reasonably well-informed about IQOS use and research and, akin with other research, generally perceived IQOS to be less harmful than smoking [14–16] although there was great uncertainty [15]. Despite these views, our analyses further identified that harm perceptions are nuanced and that users and ex-users perceive there to be some harm from using IQOS. These views were similar for perceived harm to the user as well as non-users exposed to second-hand emissions.

Consistent with qualitative research using focus groups from South Korea [31], we found that improvements in physical health and the reduced smell of IQOS led to perceptions of reduced harm relative to smoking, while the presence of tobacco in HEETS led to perceptions of some harm. Also building on our previous findings regarding what influenced use of IQOS [33], our analyses identified that perceptions of reduced harm were influenced by trust in UK market regulations, the process of heating rather than burning tobacco and the lighter nature, smell, and appearance of emissions and the appearance of used filters, in addition to PMI marketing claims and attractive HEETS packaging with less severe and specific health warnings. These perceptions were tempered by mistrust of the tobacco industry, perceiving nicotine as harmful, and residue deposits inside the IQOS device, as well as PMI disclaimers, the lack of independent and longitudinal research, and written warnings on HEETS packaging [33].

We also found that the lighter nature and appearance of IQOS emissions and PMI’s claims regarding the absence of second-hand smoke influenced current and former users’ perceptions of reduced harm to *non-users exposed to the emissions* relative to smoking, as well as reduced smell of IQOS akin to a qualitative study from South Korea [36]. However, reservations were expressed due to mistrust of the tobacco industry and lack of independent and long-term research. Perceived harm to non-users from second-hand emissions, including not only co-residents/family but also pets and members of the public, is rarely explored in relation to alternative tobacco and nicotine products; however, our data suggest that it may influence people’s decisions to use IQOS instead of combustible cigarettes, particularly around family members [33], similar to qualitative findings from focus groups [31].

This study helps to understand how individuals conceptualise harm. A reduction in harmful chemicals produced compared to cigarette smoking is a key marketing feature of IQOS [3, 37, 38], and our data suggest consumers are highly receptive to this and interpreted it as less harmful, healthier, or safer. Notably, participants specifically recalled manufacturer claims of reduced harm as a percentage relative to smoking (e.g. 90–95% less harmful). Quantification of harm relative to smoking has been used by UK public health bodies to communicate the relative harm of e-cigarettes [39]. Aligning with research on smokeless tobacco [26], our findings suggest that quantification of harm as a percentage is memorable and importantly did not appear to undermine perceptions of absolute harm; it is thus likely to be useful for communicating magnitude of risk.

Akin with quantitative studies in the UK and internationally [20, 40, 41], IQOS health harms were also conceptualised in terms of specific smoking-related diseases, including cancers and cardiovascular diseases, as well as the potential to cause new or unknown diseases. Packaging, physical and sensory experiences, the process of heating, and HEETS contents were key influences on these perceptions. First, consistent with research on other tobacco products [26–29, 42, 43], attractive HEETS packaging (Fig. 2) led to some perceptions of reduced harm relative to smoking, although there was overall uncertainty because of the lack of graphic or specific warnings. Second, similar to qualitative research on e-cigarettes [44], some participants felt that their lungs were less heavy and that IQOS use had a less negative impact on the throat and mouth compared to smoking. Third, unlike burning tobacco, the process of heating tobacco was perceived to avoid the production of tar, carbon monoxide, and carcinogens, and hence the diseases that these substances cause; however, the unknown products of heating tobacco led to uncertainty about the specific diseases caused. Fourth, some participants perceived that the tobacco, chemicals, and nicotine in HEETS must increase the risk of disease and damage to the lungs, while some considered that the unknown contents of HEETS could cause new or additional diseases from those caused by smoking.

Our finding that nicotine is perceived to be a source of harm, specifically cardiovascular disease and addiction, is consistent with prior research [20, 21, 40, 41, 45]. Some participants were concerned that their nicotine intake and potential associated health harms had changed when using IQOS compared to smoking. While it is primarily the nicotine in tobacco which is addictive, the vast majority of the harms associated with tobacco use are caused by other constituents of tobacco smoke that are generated through combustion [46, 47]. The ongoing discrepancy between the actual and perceived harms of nicotine use [20, 21, 40, 41, 45] even within our reasonably well-informed sample, highlights the enduring need for increased awareness and education of nicotine health harms, particularly because inaccurate perceptions may deter some smokers from switching to other less harmful forms of nicotine use [41].

To our knowledge, this is the first study to explicitly explore what consumers wanted to know about the health harms of IQOS use. Consistent with qualitative research on e-cigarettes [44], our sample overwhelmingly wanted clarification about the harms of IQOS to both users and those exposed to IQOS emissions, including the accuracy of PMI’s claims about reduced risk of harm, specifically from trusted sources underpinned by independent research. While participants also welcomed more conclusive research about the long-term health harms, they recognised that this will take time. Their more immediate desire was for clarification, such as about possible harms linked to HEETS ingredients, nicotine content, and IQOS emissions. Participants wanted this information to be supplied by a range of sources and in more accessible forms, possibly via written information on HEETS packets. However, these desires must be taken into consideration alongside historical evidence that labelling of ventilated cigarettes as ‘light’, ‘low-tar’, and ‘low-nicotine’ led to inaccurate beliefs of reduced harm so such descriptors have since been banned [48], and that nicotine intake depends on topographical factors as well as content. Overall, our findings suggest that current and former IQOS users want accurate information about the current state of evidence regarding IQOS harms in order to make informed decisions about their use, including whether to use IQOS around non-users and non-smokers.

Our findings have implications for the communication of IQOS harms and how to regulate HTPs. Our sample of current and former users wanted information and regulatory changes that would allow them to make informed decisions about using IQOS and around whom. Quantification of risk as a percentage relative to smoking [49] as well as the risk of developing specific diseases, will be important for tobacco and nicotine users considering their options. Manufacturer claims of reduced risk should be independently reviewed and restricted to only those found to be accurate descriptions of the state of evidence, including where there is little evidence or uncertainty. While most participants received information about IQOS harms from PMI via their website, IQOS stores, and IQOS sales representatives, this was largely due to a lack of available independent information from other sources and there was scepticism to trust PMI’s claims. Independent information and research into the harms of IQOS and heating tobacco should be readily available for tobacco users via independent sources (i.e. not the tobacco industry, such as the UK National Health Service (NHS), cancer and research charities) as it becomes available. Such information could be available online, and written and verbal information could be provided by vape shops and other retail outlets that are marketing and selling IQOS and/or HEETS, in countries where this is permitted. In the absence of specific and pictorial health warnings, labels detailing what is known about harms relative to smoking could be added to HEETS packaging to mitigate uncertainty and provide more information. More broadly, public health campaigns and messaging efforts could focus on disseminating accurate knowledge of the harms of nicotine relative to smoking.

Our study has several limitations. Our sample were all adults under the age of 60 years in one UK city (London) and had currently or formerly used IQOS. Most were, or had been, users of tobacco or nicotine for at least 6 years. Moreover, unlike typical cigarette smokers in the UK [50], most were in managerial or professional occupations. As such, our findings may not be generalisable to other groups of tobacco users, or non-smokers.

Despite these limitations, this is the first independent qualitative study of the harm perceptions of IQOS use in the UK. It stems from a wider study exploring IQOS use among smokers and ex-smokers, in which harm perceptions were identified as a key influence on motivations to use IQOS [33]; exploring the provenance of such perceptions is thus important. It provides a novel and important contribution to the field by including what individuals still wanted to know about the health harms of IQOS. Furthermore, strengths of our study include that it was informed by consultations with experts and a panel of smokers and ex-smokers, the use of multiple recruitment methods to access a sample with a diverse range of views and experiences and the use of one-to-one interviews (unlike other qualitative research in this area which has used focus groups [31]) to explore individuals’ perceptions in-depth.

## Conclusion

Our sample of current and former IQOS users generally perceived IQOS to be less harmful than smoking but not risk-free, although there was great uncertainty. Their views were similar for perceived harm to individual users, as well as non-users exposed to second-hand IQOS emissions. Reflecting feelings of uncertainty and a desire for independent and accessible information, participants wanted clarification about IQOS harms, specifically related to HEETS ingredients, heating tobacco, and emissions to others.

## Data Availability

The data analysed in the current study are not publicly available due to participant confidentiality and because we did not seek participants’ consent to share their interview data.
